# The impact of a pandemic on dental professionals' work and personal lives: A qualitative study with implications for primary healthcare workers

**DOI:** 10.3389/fpubh.2022.963410

**Published:** 2022-09-01

**Authors:** Rasmieh Al-Amer, Lucie M. Ramjan, Della Maneze, Omar Al-Rashdan, Amy R. Villarosa, Yenna Salamonson, Ajesh George

**Affiliations:** ^1^Isra University of Jordan, School of Nursing and Midwifery, Amman, Jordan; ^2^Western Sydney University, School of Nursing and Midwifery, Penrith, NSW, Australia; ^3^Australian Centre for Integration of Oral Health (ACIOH), Liverpool, NSW, Australia; ^4^Ingham Institute for Applied Medical Research, Liverpool, NSW, Australia; ^5^South Western Sydney Local Health District, Multicultural Health Service, Liverpool, NSW, Australia; ^6^Maxillofacial Jordan Medical Services, Amman, Jordan; ^7^University of Wollongong, Wollongong, NSW, Australia; ^8^The University of Sydney, School of Dentistry, Camperdown, NSW, Australia

**Keywords:** biosafety, dentists, epidemic, corona virus, SARS-CoV-2, COVID-19, primary health care, healthcare personnel

## Abstract

During a pandemic, dentists face enormous challenges due to restrictions placed on their practice and the need to comply with biosafety measures. This study aimed to explore the impact of the COVID-19 pandemic and infection control measures on dentists and their practice in Jordan and the global implications for other primary healthcare workers. A qualitative exploratory study employing face-to-face or telephone interviews, was conducted with ten dentists from the 9^th^ May to 20^th^ September 2020. An inductive thematic approach to analysis was used identifying three themes, each with two accompanying subthemes: (1) Response to COVID-19 pandemic: (1a) Government response and (1b) People's response; (2) The effects of the pandemic and response measures: (2a) Impact on work and practice and (2b) Impact on personal and social life; (3) The unanticipated gains: (3a) Altruism and (3b) Leadership and change. Stringent infection control measures were implemented to slow the spread of the virus, however limited government support made implementation unsustainable and caused financial hardship. Lack of clear guidelines, changes in practice, social distancing measures, and altered social interactions, adversely impacted daily life, triggering mental distress. Misinformation influenced response to COVID safety measures. Despite the negatives, working during the pandemic reaffirmed dental professionals' roles and purpose, with strong leadership boosting morale. Education, adequate biosafety resources and clear guidelines or policies to support and sustain stringent infection control procedures are crucial in ensuring that measures are implemented to meet the safety requirements of the pandemic response. Promoting the well–being of the healthcare workforce is equally important. Finally, altruism and strong leadership among healthcare workers can contribute to a meaningful and humane pandemic response.

## Introduction

Since the first case of novel coronavirus infection (COVID-19) in December 2019, the causative virus has rapidly spread, wreaking havoc worldwide straining public health resources (1). With an incubation period of 1 to 14 days, the virus, SARS-CoV-2, mainly affects the upper respiratory tract and is transmitted through saliva, droplets or aerosols at close personal contact, and through fomites (2). Of concern is that mutations that the virus undergo over time make the pandemic harder to track and control (3). This is exemplified by the emergence of new variants, the *Delta* variant and lately, the *Omicron* variant which has higher transmissibility, resistance to COVID-19 vaccine and higher immune escape potential, reducing population immunity (4). Consequently, the COVID 19 epidemic continues to be a public health concern almost 2 years since its first appearance.

Undoubtedly, healthcare workers are at a higher risk of contracting SARS-CoV-2 than the average population due to their proximity to individuals infected with the disease (5). Therefore, WHO has initiated a wide range of additional preventive measures, including the use of personal protective equipment (PPE), to protect them from contracting the disease (6). However, some healthcare workers have been identified as being at a higher risk than their counterparts, particularly those who work at the frontline of healthcare like nurses and doctors, and those who work with aerosol-generating procedures, such as dentists. Dentists work at very close proximity to patient's oral cavity where the viral load is highest and are also exposed to droplets, saliva and blood during dental care treatment. In addition, their work involves aerosol-generating procedures as part of daily professional practice. Considering that SARS-CoV-2 particles are able to survive in a small droplet in the air which could last for several hours, dentists are at a higher risk of being infected with SARS-CoV-2 and transmitting the infection across patients in the dental setting (7). Hence, they have been required to take more stringent measures to ensure protection and prevention of cross-infection by using filtering face-piece respirator masks in addition to other protective gears (6). These measures had a profound impact on their clinical practice.

The few studies undertaken in this area have highlighted the enormous challenge dentists and other healthcare professionals are facing due to restrictions in scheduling elective cases, treating only emergencies, booking fewer appointments, and having less revenue (7–9). For example, medical personnel are required to be fully equipped with PPEs, which are expensive and uncomfortable, leading to an increase in expenditures that will, undeniably, affect patients, and increase the treatment cost burden on healthcare systems (10). Notwithstanding scholarly reports stating that the potential risk of transmission to family members, relatives, and other patients has led to a wide range of psychological reactions among many healthcare workers including fear, stress, anxiety, burnout and depression (11).

In Jordan, case zero of SARS-CoV-2 was confirmed on the 2^nd^ of March 2020. Since then, there have been 806,501 infections and 10,542 coronavirus-related deaths reported in the country (12). The government implemented drastic measures early to contain the virus, such as complete lockdown including closure of clinical practices, activation of emergency laws, and shifting of the whole education system online. Dentists received strict measures that are in line with the international recommendations, such as restriction of dental treatment only to emergency cases and use of PPEs. The infectivity and pervasiveness of the infection, coupled with these draconian pandemic control measures impacted on the mental health of many healthcare professionals in Jordan causing high levels of fear, stress, anxiety and depression (13). However, few studies have explored their perceptions being in the frontline of the pandemic health response. To address this gap, this study was undertaken to explore the challenges faced by primary healthcare workers in the face of the COVID-19 pandemic and the impact on their professional as well as their personal lives in Jordan. As frontline healthcare workers such as nurses and doctors were under immense pressure, this study approached dental healthcare professionals for interview who had also been severely affected by the restrictions and whose practices had been closed or limited during the pandemic. Highlighting the impact of the pandemic on healthcare professionals through the lens of dentists will provide valuable insights to inform policies and practices governing the healthcare workforce in the primary healthcare sector. There is a need to explore how dental professionals view the pandemic and the government's response, how they comply with biosafety measures and the impact of restrictions on their practice and personal life, as well as their preparedness to transition to the “new normal” post-COVID.

## Methods

### Design

A qualitative exploratory approach (14) using semi-structured interviews was adopted for this study. Qualitative studies that utilize an inductive approach do not lend themselves to a priori theorizing or expanding upon the existing body of knowledge (15). To illustrate, the paucity of empirical research regarding the impact of COVID-19 on dental practices necessitated an exploration of the issue through a qualitative approach. Reporting of this paper followed the 32-item checklist of the consolidated criteria for reporting qualitative research (COREQ) (16).

### Setting

This study targeted dental healthcare professionals working in the private, military and government sectors. All interviews were conducted between May 9, 2020 and September 20, 2020. During this period, the number of COVID-19 positive cases increased from 672 (9 deaths) to 4779 (30 deaths). Many primary healthcare professionals including community nurses and physicians were seconded to support the frontline workers in response to the pandemic. Dental practices were closed or had limited operation which provided the researchers an opportunity to invite dental professionals to an interview for this study.

### Sampling and recruitment method

Using a snowball sampling technique, Jordanian dentists practicing in the capital city of Amman were invited. Following approval from the University ethics committee, information about the study was posted on the University's websites and social media platforms to recruit eligible participants for the study. Inclusion criteria were practicing dentists who: (a) completed a bachelor's degree in dentistry; (b) were registered with the Jordanian Dentists Association (JDA); (c) were working in a clinical setting in Jordan; and (d) consented to be interviewed for the study. Dental students and dental assistants and those not working in the clinics during the pandemic were excluded from the study. Those who indicated an interest were then requested to provide written consent and their contact details.

### Ethical considerations

Ethics approval was granted by the Isra University Human Research Ethics Committee (JS/BA/94), Jordan. During the interview process, in addition to obtaining written consent, participants were also informed verbally that their participation was voluntary, and they could stop the interview or withdraw from the study at any time without penalty. Permission was sought to audio-record, and all participants approved the recording of the interview.

### Data collection

The interview was conducted face-to-face or by telephone, depending on participant preference. Face-to-face interviews, lasting approximately 30 to 45 min, were conducted while adhering to the recommended infection control precautions (social distancing, handwashing and sanitizing, and wearing a mask when social distancing was not possible). Two native Arabic-speaking researchers (RA, & OA), who were also proficient in the English language conducted all the interviews. The first researcher has nursing and doctoral qualifications, while the second is a dental specialist, both are current practicing health professionals in their field and experienced in conducting interviews for research studies. Having a culturally and linguistically congruent female and male interviewers in the research team enhanced the interview and data collection process. Using the semi-structured interview guide developed by the team, data collection was initially piloted with two participants. Modifications were then made to the interview guide, following discussion with a dental practitioner on the team, as both participants discussed a number of technical issues related to dental practice (Table 1—Interview Guide). During the data collection period, both researchers kept field notes reflecting on the interview process and the challenges encountered and scheduled ongoing meetings to discuss if modification of the interview process was needed.

**Table 1:** Interview guide.

1. In relation to Jordan, what do you think of the country's response to the pandemic? 2. What do you think of the current movement restrictions? 3. How do you feel about the restrictions on dentists and on your service? 4. What has been the impact of this restriction on your dental practice, professional and personal life? Give examples. 5. During this restricted movement period: a. How did this affect your work practices and workload? b. What were the challenges and how did you overcome these challenges? 6. Are you receiving support from the health service or from your professional association? If so, please elaborate ***Transitioning back to the “new normal” dental practice*** 7. If the restrictions are lifted in the next month and a vaccine is still not available, what do you think is required to practice safely? Discuss personal safety for yourself and for your staff. 8. What alternate ways do you have to mitigate any emerging risks if there is a shortage of available equipment? 9. Based on your current experiences, how can you influence policy development for dental practice in Jordan, to be better prepared for a pandemic response in the future?

No third party was present during the interview between the researcher and participant. The interview usually began by asking each participant to recount their experiences of the restricted movement order issued by the Jordanian governmental authority at the beginning of 2020, and how this order affected their dental practice. During the interview, participants were encouraged to share their feelings and thoughts about the impact of COVID-19 on them personally. Theoretical data saturation was deemed to have been reached after 10 interviews as no new information was identified.

Every participant was sent their interview transcript for member-checking and were asked to provide a pseudonym to replace their name. During this process, two participants requested that some details be removed, which were confidential.

### Translation

As all interviews were conducted in the Arabic language, audio-transcripts were transcribed and translated to the target language, English. During the translation process specific attention was given to maintaining textual and content equivalence, as well as semantic equivalence in the English language (17). All transcript translations were performed by two research team members (RA and OA), who are proficient in both Arabic and the English language and experienced in undertaking interview transcription and translation. To maintain contextual and cultural integrity as recommended (18), the translated transcripts were then reviewed by two English-speaking researchers (YS and DM) for data cleaning and readability, with the first author (RA).

### Data analysis

Following transcription of audio-recordings and translation, textual data were analyzed thematically, using the inductive approach as outlined by Braun and Clark (19). To preserve anonymity of participants, pseudonyms were assigned by a researcher (RA). Four researchers independently read all transcripts to familiarize themselves with the data. Data analysis was primarily performed by three researchers independently. This was conducted using an exploratory approach to summarize the data into different themes to provide a representative description of participants' perceptions and experiences. Each researcher familiarized themselves with the data, generated initial codes, developed and reviewed themes and sub-themes before regrouping to compare and discuss their analysis to reach consensus in the classification and naming of themes and subthemes. Researchers used Microsoft Excel Windows 10 for textual data management and documented the audit trail of their data analysis.

## Results

Ten dentists practicing in Jordan, registered with the Jordanian Dental Association consented to participate in this study. Dentists working in the private, public and military sectors were represented. Practice sizes varied from small practices of 11 to 20 patients to large practices serving more than 40 patients. Table 2 shows the demographic characteristics of study participants.

**Table 2:** Demographic characteristics of participants.

**Demographic characteristics**	***N* = 10**
Age (years), mean (range)	35.8 (28–56)
**Gender**	
° Male	6
° Female	4
**Marital status**	
° Single	4
° Married	6
Years of practice, mean (range)	11.7 (3–30)
Holds position in the Jordanian Dental Association or Medical Syndicate	3
**Specialization**	
° General dentistry	2
° Periodontist	2
° Orthodontist	2
° Prosthodontist	1
° Endodontist	2
° Maxillofacial	1
**Type of practice**	
° Private	2
° Public	1
° Both private and public	2
° Military	2
°Both private and military	1
° Not stated	2
**Number of patients seen in a week**	
° 11–20	1
° 21–30	2
° 31–40	1
° 41 and above	6
**Health status**	
° Very good to excellent health	9
° Being treated for high blood pressure	3
° Type 1 diabetes	1
**Feeling vulnerable to COVID 19 infection**	
° No	7
° Yes	3

Three themes and accompanying sub-themes were generated from the data which related to dentists' perceptions and response to COVID-19, the impact on work practices and personal life and the unexpected benefits.

### Theme 1: Response to COVID-19 pandemic—“Just right or an overkill of power?”

Participants had mixed feelings about how the government managed and responded to the COVID-19 pandemic. Initially, most of the participants felt that the early response to contain the spread of the virus was swift and appropriate. As time passed, some felt ambivalent expressing that it might have been an overreaction rather than well-considered however they justified the actions: “*It was an overkill of power a bit but maybe if we hadn't started like this we would've ended up like other countries in a big mess*” (Leen). Some participants were also perplexed by the restrictions and measures imposed on their practices, which surfaced in narratives as a lack of trust in the dental health authorities' management of the crisis. People within the community were also confused by the changes imposed on their daily life, citing COVID-19 as a worldwide conspiracy.

#### Subtheme 1:1 government response to the COVID-19 pandemic– “It was an overkill of power a bit”

Some participants believed the measures and the strict lockdown processes were timely and warranted. They felt that the initial response was crucial to containing the spread of the virus and reducing the number of cases in Jordan as illustrated by the following quote:

“*They did the right procedures in the right time; we were earlier than other countries around us but that is what made us have lesser number of cases”* (Mousa.)

However, some participants felt the lockdown procedures were not practical nor logical. For instance, some felt that the timings of curfews were unnecessary. Further they explained that it was near impossible to expect people living in refugee camps (high density areas) to adhere to the restrictions and COVID-safe practices and therefore restrictions were unjustified.

“*I don't think there was a need for the restrictions in general, there were places where people didn't even stay home even at curfew times…especially in places like refugee camps because of the high population density there, I mean these areas are overcrowded. I have been told that no one at these camps was able to follow the instructions of the curfew yet no cases of Covid-19 were reported from these places”* (Leen).

“*And the curfew from 12am till 7am was a joke. I think it has nothing to do with the Corona[virus], it was made on a political basis, and the people knew it… We shouldn't forget that there were many non-Covid-19 patients, who needed treatment too”* (Ruby).

Participants were often seeking guidance from the Jordanian Dentistry Association. Some reported when “*we were allowed to resume our practice, I was psychologically ready, and I literally followed the protocol*” (Ruby). These participants felt that they were prepared to open their practices because they had new protocols to support best practices for their own and their patients' safety. These practices included: patients making appointments by phone, treating emergency cases, ventilating rooms between cases, signing in and measuring temperatures on arrival, and donning full Personal Protective Equipment (PPE): “*I used to wear a facemask, gloves, gown and a face shield*” (Ruby).

“*I am well-prepared, and I have a set of new protocols and my clinic follows the guidelines provided by the Jordanian Dental Association”* (Haytham).

#### Subtheme 1.2: People's response to the COVID-19 – “it's a lie and conspiracy”

Most of the participants reported that the quarantine measures negatively impacted on the economy and the people's mental and physical health. The curfews often caused a sense of panic with people rushing to buy food and groceries, causing traffic congestion and lengthy queues for medicines. People were often unable to maintain social distancing in queues because of the high density and disorganization.

“*I think it should be more thoughtful because it causes heavy traffic on the day before [curfew] and the day after. For example, on Thursday, people rush to the bakeries, to the market to buy things”* (Moath).

Often the public and even some of the participants themselves, did not understand the severity of the COVID-19 virus and/or the reason for the strict measures. Therefore, the measures and restrictions were not always followed as they had “*no scientific proof* ” and simple logic did not explain the rationale behind these stringent measures. They felt the restrictions should be limited to vulnerable segments of the population like older adults or the medically compromised.

“*…if the virus is that strong, it won't be easily transmitted because the carrier of the virus will die before it can get transmitted”* (Dalida).

Misunderstandings surrounding COVID-19 led some people in the community to view being COVID-19 positive as shameful. Others felt it was a conspiracy and believed that the number of reported cases were inaccurate as well as the number of tests that were being completed each day. The participants were also concerned about the eruption of violence in the community, if people were refused treatment due to a lack of PPE: “…*it is a tribal community and they will not accept telling them we can't treat you because of the Corona[virus], they might get violent…*” (Bashar).

“*To many people, corona[virus] wasn't a disease, it was like something wrong they did and they were ashamed of it, that's not right”* (Dalida).

“*…now we don't know what's right and what's wrong, also the Secretary of Health is overestimating the procedures, he says that we did 5,000 random tests in a day! No one can do that...”* (Bashar).

“*…they say that it's a lie and conspiracy, and they didn't also care about distancing at all…our community is aggressive if someone came and didn't find a ventilator, his family would go violent and do a mess*” (Leen).

### Theme 2: The effects of the COVID-19 pandemic and response measures – “I'm afraid of being infected, for sure, but I need to live my life too”

Participants felt the impact of the pandemic and the response of the dental association to control it on their work procedures and processes as well as their personal and social life. Fear of being infected and fear of transmitting the infection to their family were grave concerns for many. As a consequence, there was increased awareness of and adherence to infection control measures in their clinical practice and reduced social contact with family and friends. However, as the pandemic continued, some participants felt that the fear of being infected cannot take over their lives.

#### Subtheme 2.1: Impact on work and practice - “I closed my clinic according to government's orders. I was afraid, to be honest”

At the beginning of the pandemic, the government responded strongly by closing many of the dental practices and restricting treatment to emergency procedures only, as dentistry was considered a high-risk practice. They also issued requirements particularly in the use of personal protective equipment and restricting procedures to the use of low-speed instruments. While many participants felt this was necessary, they also noted that there was no clear demarcation between emergency and urgent cases. Fear for their own safety inevitably was an important consideration that influenced their practice.

“*Look this is a very gray zone. A couple of days ago a doctor called asking me if this was an emergency case, she had a patient whose temporary filling fell off. He's in pain but this is not an emergency what should she do? The thing is we define emergencies as if it is any case with pain or the pain can't be managed by medicine”* (Sarah).

“*In the protocol that we should follow, we were ordered not to use high speed hand piece, but the doctors were afraid of using the low speed hand piece too, we would give the patient an antibiotic if his case can be handled this way”* (Nadeem).

Many of the participants were conflicted between providing just emergency palliative treatment in obedience to COVID-19 restriction guidelines and providing full treatment as dictated by professional ethics and best practice.

“*Yes, there were a lot of them [repeat visits], and this increases the risk that's why I thought that we should've treated them fully the first time they came. Many of my colleagues were afraid of contracting it during the curfew, so they just prescribed antibiotics and analgesics for them, even though they knew it won't help them. It was an unethical practice and those same patients would come again with worse pain or even dental abscess or swelling”*. (Dalida).

In addition, participants felt that the dental association seemed to be formulating rules without adequate support or resources. Participants were confused about what was safest to follow for themselves and for patients.

“*There is a lot of exceptions on working and prescribing medicines, there are more exceptions than laws, therefore there is no law”* (Bashar).

“*You can't work with high speed, you can't use the three-way syringe, you have to wear a face mask and you can't use the dental spittoon and it should be wrapped [talking about the restrictions imposed on dental practices]”* (Moath).

The closure and restrictions on cases that can be treated severely reduced the number of patients in practice which in consequence, radically affected their incomes. Furthermore, elective procedures were canceled or postponed and the costs of the required PPE were not sustainable in practice particularly when patients were also financially burdened. Cosmetic dentistry was a thing of the past as no patient came for treatment.

“*Some of the doctors were offered 50% percent of their salaries….and those who refused to accept the 50% were fired, because they cannot give them full salaries, but most of them accepted the 50%”* (Bashar).

- “*The patients' dental concerns changed too, most of my patients would do regular check-ups, whitening, scaling, or composite facing. None of these patients contacted me, even when we had near zero cases of corona[virus]. It's like no one cares about ‘Hollywood smile' anymore”* (Ruby).

Participants felt that the changes instigated as a response to the pandemic, although beneficial to maintain best sanitary practice, were not sustainable in the long term. These included complicated, time consuming and expensive sterilization procedures and the use of Personal Protective Equipment.

“*The general area of the clinic is sterilized by water and detergent. The transfer area we clean it, first with water and soap, then you check it if there is anything visible to the eye, if not, you disinfect it by 1% sodium hypo chloride if it's not metallic. If it is, you use aldehyde containing disinfectant, after this you get it out of the dirty zone and put it in the clean zone or autoclave. And the last zone, which is the surfaces where you treat the patient, you sterilize it by 1% sodium hypo chloride and use hard wipes to clean all the surfaces, then you wrap all the touching surfaces and when the patient finishes you unwrap them, disinfect them again, then you wrap them again with new wrapping paper, and every tool you use on a patient should be fully disinfected”* (Sarah).

As the pandemic continued, moving forward, participants felt that acknowledging the existence of the virus and adjusting to its presence seemed to be the only logical response to continue with life as it was known prior to the pandemic. However, obtaining adequate training to deal with the virus and its possible consequences and taking the right precautions, where feasible, were seen as necessary elements of a pandemic response.

“*I will take the right amount of precautions, nothing less, nothing more. I'm afraid of being infected for sure, but I need to live my life too and this virus looks like it's not going anywhere, so we just have to live with it”* (Ruby).

#### Subtheme 2.2: Impact on personal and social life - “I feel we live in an isolated zone… ”

Similar to the people in the community, participants felt the psychological impact of the pandemic and its restrictions. They described being paranoid about whether sterilization used in their clinic was adequate, or if they had inadvertently treated patients who were COVID-19 positive. One participant felt that this would destroy his reputation were it to be known in the community that a COVID-19 patient visited his clinic.

“*I fear for my family to get infected, to be honest I am afraid to open my clinic because I'm going to sacrifice my reputation that I've build over years if an infected asymptomatic patient came and I treated him, or if anyone tested positive and claimed that he/she visited my clinic, also, there is still a risk that other patients will get infected, even if the protocols are followed and the authorities will lockdown my clinic, and all my hard work through years will drain down the sink”* (Morad).

A participant was concerned about the change in the interaction between patients and healthcare professionals as this might affect the therapeutic relationship.

“*The old friendly system that we used to use with them [patients] won't be applicable anymore, we'll have to be more formal”* (Sarah).

They also expressed feelings of sadness being isolated, socially distanced and quarantined from their family and friends recognizing that culturally they are a people who valued human relationship and expressed this physically by hugging and being friendly. One participant described this:

“*You know in the normal situation we make a lot of gatherings and family dinners, we always shake hands and hug each other, it's in our blood”* (Nadeem).

While they were given passes by the authorities to freely move around in the community because of their medical profession, they reported that they only went out to buy groceries. There were many participants who overwhelmingly reported that they did not visit family and friends out of fear that their exposure to patients might have “*contaminated*” them and they would in turn, infect vulnerable family members.

“*No social gatherings at all even when my sister was sick, I didn't pay her a visit. I was scared that she could have caught the virus from me (although I did not have any symptoms, but you know, I deal with many patients). Also, people didn't want me near them because I worked in a health center, so it was a mutual feeling between me and my family that we shouldn't see each other for the time being”* (Sarah).

Still others felt that they were treated differently by other members of the family, fearing that they were infective because of their profession. As one participant, whose father was a retired dentist, expressed:

“*On the personal side, I think my dad is still treating me differently [does not hug her], even till now, maybe because he is a dentist [therefore he knows the risk I am exposed to]. I don't know, but he is still afraid of me, he didn't give me a hug since the beginning of all of this”* (Ruby).

Some became more innovative in the way they reached out to family and friends using other forms of communication such as video conversations. However, younger participants, interviewed when the number of cases were tapering down, expressed less fear and were less adherent to the social distancing and quarantine measures with some feeling that there will be “*rebound socializing*” after the crisis.

- “*Yes, honestly I gathered with my friends and didn't adhere to social distancing at all, maybe it wasn't the smartest thing to do, but I wasn't afraid”* (Dalida).

### Theme 3: The unanticipated gains - “I felt I'm more than just a dentist”

From the chaos and despair in a very challenging situation sprung unexpected gains. The participants explained how the pandemic had changed them and transformed their behaviors and practices. There was a strong sense of empathy and concern for the people in their community and a need to rally together and support each other at this tumultuous time, despite the financial and emotional burden. While there were mixed feelings on leadership styles at the peak of the pandemic, many highlighted that they appreciated those who stepped up and provided strong leadership. This better prepared them for the future.

#### Sub-theme 3.1: Altruism was second nature – “you had a noble purpose to work 24/7…”

The Jordanian community is known as a collectivistic society, prioritizing the needs of others over their own individual desires. These social values and focus on the community's well-being were heightened during the peak of the pandemic. Participants admitted that they went out of their way to ensure that they could get to their work practices to service the community and their dental needs; some car-pooled with others as they had no other means of transportation at this time. Their main concern was to provide oral healthcare.

“*There was no transportation method for me to go to work, so I used to meet up with my friends every day and go to work together. It wasn't that hard but still, we got stopped by police a couple of times, and we showed them that we are dentists”* (Dalida).

One participant, a female dentist, described how she drove fifty kilometers (round trip) each day to pick up a nurse who worked in her clinic. The pandemic seemed to bring out the best in people.

“*Most of us were females, especially the nurses, I even used to pick up a nurse from her home to work and bring her back, I drove about 50 kilometers a day, and it wasn't disturbing at all. I was very happy to do it”* (Leen).

These acts of altruism were common, with most of the participants reporting how they or others volunteered to do more and sacrificed more, including their own health and financial security “*for the greater good*”. Many could not raise prices for services even though they were facing mounting economic burden.

“*The price raise for each patient is about 12–20 JDs, but you can't raise this price for the patient, because he is also in financial problems like us, now we work not for the profit but for the country for each other to help each other, it's not humane to raise the price during this time”* (Morad).

One participant commented that as a consequence of the pandemic, she now felt gratitude and a greater appreciation for the work she does as a dentist. There has been a shift in reference points in terms of what makes people happy. Despite the loss of income for many and the long hours of work, participants were more satisfied in their jobs because they were connecting with the community on a more meaningful level, rather than a fee for service.

“*Actually, while the crisis was at its peak, I was happier and felt like I'm a human and more comfortable dealing with patients, because back then it was like you had a noble purpose to work 24/7… me it was more than just a job, it was taking a part in something greater and helping the people in need, I felt like I'm more than just a dentist. I felt like a hero working for the greater good”* (Leen).

#### Sub-theme 3.2: Leadership and change – “he brought the best out of everyone around him”

What was evident was the need for strong, robust leadership and role-modeling during the pandemic. This type of leadership style “*brought the best out of everyone*” (Leen). There were mixed reviews on the leadership in different settings across all health sectors (private and public). One participant was particularly dissatisfied with the leadership and management and felt that some of the specialists avoided patient contact by sitting in their offices, delegating their work to the residents. This participant highlighted the importance of seniors being better role models for others in a pandemic.

…*the head of the residents his role was very unsatisfying he only managed our schedule in the clinic, but he didn't treat any patients at all as I said before some people took advantage of the situation just to work less*. … *our superiors should be more caring, and specialists shouldn't just throw all the workload on residents* (Dalida).

Despite the above, there were examples of exemplary leadership during adversity. One example showcased how the Director of a dental clinic stepped up and took charge. He went above and beyond to source volunteers from the community, using his connections, to assist him with raising donations for medicines and then distributing those medicines to people in need. He led his staff by example and was strict in maintaining the highest standards within his dental practice. He protected his community as a father protected his children.

“*We acted like a true family, and the Director did an outstanding job. He is a true leader, he brought the best out of everyone around him, and in that period that was exactly what we needed. He raised donations for those in need, like those patients who couldn't pay for their medication, he also gathered lots of volunteers who came to help, because we had at least 60–70 people queuing in line while in the crisis waiting for their medicine. It was a lot of pressure. People were afraid of medicines running out”* (Leen).

Overall, the pandemic contributed to a reassessment of priorities. For some they have realized the importance of meaningful connections with community and family over wealth - “*The crisis taught us something, that working less produces more, there is no need to work 8 continuous hours”* (Bashar). Further COVID-19 instigated a positive change in dental professionals' ways of thinking and working. The pandemic has been a wake-up call. They believed they were better prepared to maintain and respond to public health issues post the crisis and will continue to uphold these best practices, including the donning of PPE, for the health and safety of the community and themselves.

“*I think we should keep wearing the same protective gear even when this crisis is over. I think this a big reminder for all health care providers*” (Haytham).

## Discussion

While this qualitative study explored the impact of the COVID-19 pandemic on dental professionals who were working in Jordan, our findings resonated with experiences of many healthcare workers globally (20, 21). Participants in this study confirmed that they had to modify their practices upon re-opening of clinics, and re-enforced the need to adopt stricter sterilization procedures, personal protective measures and screening protocols to maintain their own and the public's health and safety (9, 22, 23). As with many healthcare workers, dentists are required to be in close proximity with patients, which places them at high-risk for contracting the infection through dental aerosols and respiratory droplets during procedures (8). It was, therefore, not surprising, that routine dental services were suspended, and care was restricted to emergency cases only (24). However, dental professionals in many parts of the world were not prepared for the restrictions imposed on their practice (25, 26) and those interviewed in this study were no exception. Consequently, they were confronted with many challenges including uncertainties about the distinction between emergency, urgent cases, and non-urgent cases. Participants expressed indecision regarding cases they were allowed to treat, particularly in the absence of clear guidelines from their own professional association and seemingly *ad hoc* rulings from authorities which confused rather than clarified. In addition, they also faced the moral dilemma of extending only symptomatic treatment to non-emergency cases when this was clearly not aligned with professional guidelines (27). They had the additional responsibility of considering the collective welfare of the public, their own safety and balancing this with providing definitive treatment for all patients as required by their professional ethics (28). Unlike dental health workers, many primary healthcare workers continued to work during the pandemic. However, congruently, they also had to contend with changes in their practices including retraining and redeployment to cope with the rapidly increasing number of positive COVID-19 cases (20). This is particularly true for frontline nurses who must adapt to the increased workloads, discomfort with extra layers of PPEs and greater risk of exposure to occupational hazards, including contact with contaminated instruments and patient secretions, contributing to higher anxiety not only for their own safety but that of their families (29).

Compounding these are the perceptions and beliefs derived from misinformation in different forms and platforms from all sectors of society (30). In our study, the perceptions and beliefs among dental professionals and the public related to COVID-19 being a conspiracy was also confirmed in a cross-sectional study among the general public in Jordan which identified that almost half (47.9%) believed COVID-19 was a global conspiracy while over four-quarters (82.7%) believed that it was a test or trial from God (31). Similarly, a study in Ecuador among healthcare workers showed that those who believed in the conspiracy theory were more likely to experience anxiety and psychological distress (32) and were identified to have higher vaccine hesitancy (33). Lack of knowledge and stigma can invoke fear and aggression (34), highlighting the importance of authorities providing factual, evidence-based, and consistent communication in a crisis.

While restrictive measures may have mitigated the caseload of the virus, the measures instituted impinged on the personal freedom of people (35). Research reports that isolation and quarantine measures can cause increased stress, anxiety and depression (36), and this was a concern reported by participants in our study. The greatest fear and anxiety for dentists during the pandemic was the possibility of inadvertently acquiring and transmitting the virus to other patients and to their own families which was similar to the findings of a large study involving dental professionals across 30 different countries (11). Social distancing measures during the pandemic, also impacted on family relationships. Family members were fearful that they may harbor the COVID-19 infection (37). This had serious mental health effects and should be considered in future interventions. Similar findings of depression, anxiety and distress were reported among other Jordanian nurses and doctors, further reinforcing the need for more psychological support for frontline healthcare workers during a pandemic (13).

Of final note, given that Arabic culture is collectivist, where social life is family-centric rather than egocentric and common good transcends individual interests (38), the collectivist nature of the Jordanian dental professionals in our study was clearly present. Altruism and overall strong leadership in a crisis surfaced during the turmoil. In most settings, many went above and beyond to mitigate the social, economic and financial impact of the pandemic through advocacy and volunteerism. Many were adamant that they were better prepared and would continue with personal protective measures and biosafety practices within their clinics after the pandemic which further emphasizes the strong collectivist values and desire among Arabs to protect others in the community (39). Therefore, future guidelines and protocols published by dental associations and other healthcare organizations for use during a pandemic need to consider the collectivist orientation and strong family ties within communities to ensure the appropriateness of restrictions, resulting in less fear and greater acceptance of changes to practice. While this study considers dentists as primary healthcare workers who like others in the health workforce who are required to work in close proximity to patients, the transferability of research findings to other primary healthcare workers may be limited because of inherent differences in roles and responsibilities.

The sample size of the study, while considered small, was determined by achievement of theoretical data saturation and pragmatic considerations during the pandemic. However, a strength of this study was ensuring that dental practitioners from different sectors of practice, including private, public and military were included. As recruitment of the sample was done through convenience and purposive sampling, this may have resulted in sample selection bias.

### Relevance to clinical practice

Considering that this is a small qualitative study, the findings provide rich insights into the impact of the COVID-19 pandemic at its peak, on primary healthcare workers who work closely with patients. Our study provides researchers an opportunity to confirm these perceptions in similar studies with other healthcare worker subgroups. Of note from this study is the impact of misinformation and rumors which can further increase mental health distress among the community and within an already strained healthcare workforce. Practice implications from this study include the need for frontline health staff and the general public to have effective leadership and access to trusted sources of information to remain informed. Further infection control policies, guidelines, screening tools and protocols for various healthcare professionals should be standardized and based on international benchmarks, including consistent public health orders, such as the importance of mandatory vaccinations for all workers in the health system. To avoid risk of exposure and further psychological distress, sufficient funding and resource allocation of personal protective equipment is necessary to sustain stringent infection control measures and maintain safety in practice. Finally, in view of the collectivist culture in Jordan and the impact of restrictions such as social distancing on mental health, there is a need for access to greater mental health support interventions and services for both healthcare workers and the public in a crisis.

## Conclusions

The pandemic and requisite control measures adversely impacted the professional dental practice and the personal life of dentists working in Jordan. Effective leadership and positive role-modeling of best biosafety practices sustained staff morale and confidence in often challenging working environments. Fear, anxiety and stress are natural responses to the pandemic particularly one as pervasive as COVID-19, however, it is imperative upon health authorities to provide enhanced mental health support for healthcare workers in the frontline of the pandemic response.

## Data availability statement

The data supporting the conclusions of this article will be made available by the authors upon reasonable request, however, pseudonyms will be used to protect the identity and privacy of participants.

## Ethics statement

The studies involving human participants were reviewed and approved by Isra University Ethics Committee [no (JS/BA/94)]. The patients/participants provided their written informed consent to participate in this study.

## Author contributions

RA-A, LR, AG, OA-R, AV, YS, and DM were responsible for the study conception and design, were responsible for drafting the manuscript, and made critical revisions to the paper for important intellectual content. RA-A and OA-R collected and translated the data. DM, RA-A, and YS collated and prepared the data. LR, DM, OA-R, and RA-A performed the data analysis which was validated by YS, AG, and AV. All authors made substantial contributions to the study and fulfilled the definition of authorship set up by the International Committee of Medical Journal Editors (ICMJE).

## Funding

This work was supported by Western Sydney University and the Australian Centre for the Integration of Oral Health (ACIOH).

## Conflict of interest

The authors declare that the research was conducted in the absence of any commercial or financial relationships that could be construed as a potential conflict of interest.

## Publisher's note

All claims expressed in this article are solely those of the authors and do not necessarily represent those of their affiliated organizations, or those of the publisher, the editors and the reviewers. Any product that may be evaluated in this article, or claim that may be made by its manufacturer, is not guaranteed or endorsed by the publisher.
